# ASO Author Reflections: Combining Two Easily Available Parameters Can Help to Individualize Lymph Node Surgery in Medullary Thyroid Cancer (MTC) Without Compromising Cure Rates

**DOI:** 10.1245/s10434-025-17088-0

**Published:** 2025-02-28

**Authors:** Martin B. Niederle, Teresa Binter, Philipp Riss, Bruno Niederle, Christian Scheuba

**Affiliations:** 1 Division of Visceral Surgery, Department of General Surgery, Medical University of Vienna, Vienna, Austria; 2 Department of General Anesthesia, General Intensive Care and Pain Management, Medical University of Vienna, Vienna, Austria

## Past

Regardless of size, medullary thyroid cancer (MTC) spreads very early to lymph nodes (LNs).^1^ Lymph node metastases (LNMs) negatively affect long-term prognosis.^2^ Definite cure of MTC can be achieved by early disease diagnosis and subsequently by appropriate early stage surgery, ideally before LNMs develop.^3^

Timely diagnosis is achieved by consistently measuring basal (b) calcitonin (Ct) during the diagnostic workup of thyroid nodules, as Ct is the most important biomarker for MTC diagnosis.^4^ Compared with bCt, the sensitivity of ultrasound and functional imaging (fluorodopa-positron emission tomography-computed tomography [F-DOPA-PET-CT]) is low in detecting either small primary tumors (in particular) or central and lateral micro-LNMs.^5^ Assay- and sex-specific bCt levels have been shown to correlate well with tumor mass (size, extent of LNM, and distant disease [M1]).^2,6^

To avoid missing “occult” LNMs in the central and lateral neck, current guidelines have summarized clear general recommendations on the extent of initial surgery: for patients with suspected, localized, regionally “clinically apparent” MTC (T1b–4 N0/1), for those with “micro” MTC (10 mm or smaller; T1a N0/1), and for patients with distant metastasis, thyroidectomy, bilateral central neck dissection (CND), and in select patients, unilateral neck dissection (ULND) or bilateral lateral neck dissection (BLND) have been advised.^7^

Patients who were not operated on according to these recommendations were shown to have shorter survival rates than those who underwent surgery in line with the abovementioned recommendations.^8^

However, when choosing the optimal surgical strategy, the oncological benefit must be weighed against the risk of surgical complications, as extensive but not necessarily indicated surgery also increases the risk of complications.^9^

Recently, it was shown that both well-defined pretherapeutic bCT cutoff levels^2^ and the intraoperative distinction between desmoplastic stroma reaction (DSR)-negative and DSR-positive tumors by frozen sectioning (FS) can predict the presence of LNMs.^10^ On the one hand, subdividing patients into risk groups according to their pretherapeutic bCT allows for the determination of the probability of LNMs (“oncologic risk”);^2^ on the other hand, DSR negativity is always associated with the absence of LNMs (and M1).^10^

To date, the combination of both parameters has not been analyzed for its potential to individualize (tailor) the extent of LN dissection (besides thyroidectomy) without compromising cure rates.

## Present

On the basis of 30 years of experience with a Ct screening program, in which bCt was routinely measured in all thyroid nodules during diagnostic work-up, biochemically suspected and histologically verified (sporadic and hereditary [index]) MTCs were analyzed in 306 patients.

A uniform surgical protocol (thyroidectomy, bilateral central neck dissection [CND], and lateral neck dissection [LND]) was applied in all patients.

FS was applied intraoperatively, and the primary tumors were divided into DSR-negative and DSR-positive lesions.

Pretherapeutic bCt, intraoperative FS, and definitive histological results of the primary tumor, number of removed and affected LNs, and biochemical and clinical follow-up data were retrospectively correlated.^11^

Using predefined sex-specific cutoffs, 115 patients were assigned to the “minimal-risk” group (defined as: female patients, bCt ≤ 23 pg/mL; male patients, bCt ≤ 43 pg/mL), 50 to the “low oncological risk” group (female patients: 24–84 pg/mL; male patients 44–99 pg/mL), and 141 to the “high oncological risk” group (female patients: ≥ 85 pg/mL; male patients ≥ 100 pg/mL). “Purely” DSR-negative tumors were verified in 63/306 patients (20.6%). Central LNMs were found in 3/115 (2.6%) “minimal-risk” patients and in 3/50 (6%) “low-risk” patients. Patients with lateral LNMs (72/141; 51.1%) were documented exclusively in the “high-risk” group. Regardless of their assignment to the pretherapeutic bCt risk groups, all patients with intraoperatively documented (always confirmed postoperatively) DSR-negative tumors were N0 (and M0) and all (100%) were cured during long-term follow-up.

The cure rate for patients in the “minimal” and “low oncologic” risk groups with DSR-positive tumors was 98.2%. Bilateral lateral LNMs were not identified in “high-risk” patients (DSR-positive) with bCt ≤ 350 pg/mL (*n* = 41). In this subgroup, the cure rate was 35/41 (85.4%). When bCt exceeded > 350 pg/mL, only 39/68 (57.4%) were cured. All patients with bilateral lateral LNMs (*n* = 8) showed persistent disease.

## Future

The extent of surgery, such as CND and/or LND, can be individualized by applying two easily available and economical parameters, thus minimizing surgical trauma and reducing surgical complications without compromising cure rates in patients with biochemically suspected MTC: the routine assessment of pretherapeutic bCt in combination with the consistent application of FS, discriminating intraoperatively between DSR-positive and DSR-negative MTC. Thyroidectomy should remain the standard of care, as any subtotal thyroid surgery may fail to cure MTC owing to the high rate of multifocal bilateral disease (26%), even in sporadic MTC. In at least a quarter of patients with sporadic and hereditary (index) MTC, any lymph node surgery can be omitted if “purely” DSR-negative tumors are documented intraoperatively. In DSR-positive lesions, the extent of LN dissection can be tailored when assigning patients to the specific, well-defined pretherapeutic bCt risk groups.

Currently, no biomarkers are available to definitively predict LNMs in the preoperative setting. Additionally, fine-needle aspiration (FNA) biopsy applied to make use of molecular markers in the tumor sample to detect a more aggressive MTC variant preoperatively must be avoided. This is because the needle may traumatize the tumor, potentially triggering the development of scar tissue with a misinterpretation of DSR positivity in FS.^12^

The intraoperative assessment of the mitotic and Ki67 proliferation index (grading) of the primary lesion (although potentially helpful to predict long-term prognosis) does not seem practicable, as immunohistochemical reactions, as well as mitotic cell counting, are time-consuming techniques. Moreover, even “low grade” does not exclude LNMs in advance.^13^

Future prospective research should focus on verifying this simple yet effective concept of combining pretherapeutic bCt and intraoperative DSR. In addition, the applicability in detecting the optimal time for surgery and planning its extent in patients with genetically “screened” hereditary MTC (considering moderate- and high-risk genetic mutation variants) should be tested.
